# OVARIAN AGING: A TARGET FOR GEROPROTECTION IN WOMEN

**DOI:** 10.1093/geroni/igad104.0999

**Published:** 2023-12-21

**Authors:** Yousin Suh

**Affiliations:** Columbia University, New York City, New York, United States

## Abstract

The ovary is the first organ to undergo early-onset aging in the human body, affecting both fertility and overall health in women. However, the biological mechanisms underlying human ovarian aging have been poorly understood. To understand the molecular, cellular, and genetic basis of ovarian aging in humans, we performed integrative single-nuclei multi-omics analyses of young and reproductively aged human ovaries. Our analysis reveals coordinated changes in the regulatory landscapes across all cell types in the ovary during aging, characterized by transcriptomic and chromatin accessibility signatures of the canonical “Hallmarks of Ageing”. By performing integrative analyses of our single-nuclei multi-omics data with genetic variants associated with age at natural menopause from genome-wide association studies (GWAS), we demonstrate a global impact of functional genetic variants on changes in gene regulatory networks across ovarian cell types. Our work provides a comprehensive multimodal landscape of human ovarian aging and mechanistic insights into inherited variation influencing the timing of menopause. Our results raise the hope that geroprotectors targeting the “Hallmarks of Aging” may be used to delay ovarian aging, thereby promoting reproductive health and extending healthspan and longevity in women.

